# Study on the spatiotemporal variations of vegetation phenology in China based on solar-induced chlorophyll fluorescence

**DOI:** 10.3389/fpls.2026.1882899

**Published:** 2026-07-20

**Authors:** Shikang Zhang, Xiaotong Li, Wei Zhang, Chenchen Yuan

**Affiliations:** 1 College of Resources and Environment, Anhui Science and Technology University, Fengyang, Anhui, China; 2 College of Management, Anhui Science and Technology University, Bengbu, Anhui, China; 3 College of Architecture, Anhui Science and Technology University, Bengbu, Anhui, China

**Keywords:** climate change, growing season, remote sensing, SIF, vegetation phenology

## Abstract

This study analyzed the temporal and spatial dynamics of vegetation phenology using high-resolution solar-induced chlorophyll fluorescence (HCSIF) data combined with meteorological and land use data. Regression, stability, continuity, and correlation analyses were conducted to evaluate the effects of climate change on vegetation phenology in China over the past 20 years. The results showed that, first, the start of the season (SOS) ranged from early January to mid-May and the end of the season (EOS) from mid-August to late December, with an average growing season (LOS) of 8 months. Over 20 years, SOS advanced by 0.02 days/year, EOS was delayed by 0.23 days/year, and LOS lengthened by 0.13 days/year. Second, most vegetation types exhibited delayed EOS and prolonged LOS, except grasslands and shrubs, which showed slightly advanced EOS and shortened LOS. Cultivated land and natural vegetation experienced the most significant changes, with SOS advancing by 0.66 days/year, EOS delaying by 0.97 days/year, and LOS increasing by 1.37 days/year. Lastly, elevation and urbanization had notable effects: every 500-m elevation increase advanced SOS by 3.67 days, delayed EOS by 2.87 days, and extended LOS by 6.53 days; a 10% urbanization increase led to a 1.37 days/year SOS advance, 2.22 days/year EOS delay, and 3.09 days/year LOS extension. Meteorological factors strongly influenced the phenological changes, particularly in cultivated and natural vegetation.

## 1 Introduction

Vegetation phenology is a key indicator of climate change and refers to the response of vegetation to external factors (temperature, precipitation, etc.) during the growth period, including the processes of germination, flowering, fruiting, and defoliation. As an “early indicator” of the response of the ecosystem to climate change, it has substantial monitoring and prediction value in the context of global change (Richardson et al., 2018). As global climate change and human activities intensify (Huang et al., 2016; Jiang et al., 2020; Luo et al., 2018; Zhang et al., 2016), terrestrial ecosystems are changing significantly, and there is an urgent need to accurately monitor and analyze the dynamic characteristics of vegetation phenology and its intrinsic driving mechanisms to respond to the challenges and threats of climate change.

The vegetation phenology monitoring methods currently used include ground surveys (Richardson et al., 2007; Zhang et al., 2003), remote sensing (White et al., 2009), model simulations (Chuine and Beaubien, 2001; Jolly et al., 2005), and three other methods. Ground survey is a traditional method that can accurately characterize the growth and climatic status of specific plant species. However, this method is hard to apply to large-scale biomass research because of the limitations of the spatial distribution of observation stations, observation species, and coverage (Gong et al., 2024). Vegetation models can accurately simulate the spatial and temporal dynamics of vegetation under different conditions by simulating the plant growth process and its meteorological and environmental parameters and constructing ecologically meaningful crop growth models. However, owing to the limitations of the theoretical assumptions of the model, model structure, and simulation scenarios, the results of the different models are quite random and uncertain, which restricts their application in ecological management research (Xiaoying et al., 2024). With the joint efforts of researchers around the world, many remote sensing indicators, such as NDVI and EVI data, have been used to monitor vegetation phenology, which makes it possible to study the dynamics of plant phenology at large scales and in long time series. However, it is affected by multiple factors, such as cloud cover, aerosol interference, sensor band differences, and data synthesis algorithms, which cause problems such as insufficient accuracy in climate monitoring, errors in determining key climatic phases, and limited recognition ability in areas with high vegetation cover or complex terrain (Wang et al., 2018). As a complement to the NDVI, sunlight-induced chlorophyll fluorescence (SIF) based on vegetation canopy reflectance spatially provides a new solution for monitoring vegetation function. SIF is a spectral signal emitted by vegetation through photosynthesis under light conditions, and the data are insensitive to atmospheric and cloud scattering, which can directly reflect the dynamic changes in vegetation as it undergoes photosynthesis (Chen et al., 2022).

SIF provides a unique means of monitoring vegetation photosynthetic activity and physiological responses to environmental variability (Guanter et al., 2014). Compared with NDVI, SIF is more closely related to ecosystem carbon uptake and photosynthetic functioning, thereby providing complementary information for understanding vegetation responses to climate variability (Joiner et al., 2014; Sun et al., 2017). Unlike NDVI and EVI, which primarily describe changes in vegetation greenness and canopy structure, SIF provides direct information on photosynthetic activity and ecosystem carbon uptake. Consequently, phenological metrics derived from SIF represent photosynthetic phenology rather than greenness phenology (Walther et al., 2016). Leaf area index (LAI) is another widely used indicator for monitoring vegetation growth and ecosystem productivity because it characterizes canopy structure and leaf abundance (Asner et al., 2003; Myneni et al., 2002). However, LAI mainly reflects the structural attributes of vegetation and may not accurately capture short-term physiological responses to environmental stress (Goel and Qin, 1994; Steltzer and Welker, 2006). In contrast, SIF is directly linked to photosynthetic processes and can respond rapidly to changes in plant physiological activity. Therefore, SIF provides complementary information to LAI and may better characterize vegetation functional dynamics and photosynthetic phenology. Previous studies have shown that the timing of photosynthetic activity may differ from that of canopy greenness, particularly in regions affected by temperature limitation, water stress, or complex environmental conditions (Walther et al., 2018). Therefore, SIF-based phenology can provide complementary insights into vegetation functioning and carbon cycle dynamics that are not fully captured by traditional vegetation indices. For China, most existing large-scale phenological studies have relied on NDVI or EVI greenness phenology (Peng et al., 2017; Yan et al., 2015). In contrast, the high-resolution HCSIF dataset enables the investigation of photosynthetic phenology at a national scale, providing new perspectives on vegetation responses to climate variability and environmental change. With the continuous development of remote sensing monitoring satellites, satellites such as GOME-2, GOSAT, and OCO-2 provide global-scale SIF observations, but their low spatial resolution limits their application in regional or surface fine-scale analysis. High-resolution SIF data not only improve the accuracy of determining the occurrence time of vegetation phenology, such as the greening and yellowing periods, but also provide the possibility of revealing differences in the photosynthesis dynamics of different vegetation types in a region (Liu et al., 2023). HCSIF data are more sensitive than NDVI in capturing climatic changes in urban green areas, agricultural boundary zones, and ecologically fragile zones, which is particularly suitable for analyzing the vegetation response under the urban heat island effect and anthropogenic disturbances (Wang et al., 2019).

Based on multisource SIF datasets, researchers have conducted many studies on plant phenology and its driving mechanisms—for example, Zhou et al. (2021) conducted a global-scale vegetation phenology study based on OCO2 SIF data. They reported that the SIF was more sensitive to the onset of the growing season than the traditional VI and that the SIF was significantly correlated with meteorological factors such as preseason shortwave radiation and temperature. Additionally, Merrick et al. (2019) and Song et al. (2018) conducted studies on the vegetation phenology of different biomes in Brazil, the equatorial northern hemisphere, and typical regions of China, respectively, using OCO2 and GOME2 SIF data to monitor and analyze the spatial and temporal characteristics of vegetation phenology in these regions. The remote sensing monitoring results indicate that, in recent decades, the vegetation seasons in the Northern Hemisphere at middle and high latitudes have advanced in terms of the SOS during the greening period, started growing earlier, delayed the EOS during the end of the growing season, and lasted for a longer growth period (Yingying et al., 2019). The abovementioned studies show that, in the context of global climate change, the vegetation phenology in different regions and scales presents extremely complex dynamic characteristics. Revealing the intrinsic driving mechanism of vegetation phenology has become a popular research topic in the context of global and regional change. Due to global warming, the growing season has advanced, and the end of the growing season has been delayed (Vitasse et al., 2022)—for example, Wang et al. (2015) noted that the increase influences the advancement of spring phenology in terms of winter and spring temperatures. This trend is significant in temperate and high-latitude regions (Chuine and Régnière, 2017). In the Northern Hemisphere, plant phenological events advance, on average, by approximately 2.5 days for every 1°C increase in the mean spring temperature (Fu et al., 2015). A study by the Institute of Tibetan Plateau Studies of the Chinese Academy of Sciences (Li et al., 2016) revealed that warming mainly prolonged the flowering period of alpine plants, which, in turn, lengthened the reproductive period and the whole growing season, whereas cooling shortened the post-fruiting nutrient period, yellowing period, nutrient period, and the growing season. Moreover, vegetation phenology is sensitive to changes in precipitation (He et al., 2018; Ma et al., 2015). In arid and semi-arid ecosystems in the Great York Basin of the United States, changes in precipitation in spring and winter strongly affect the interannual variation in vegetation greenness. In contrast, temperature has a relatively small effect (Tang et al., 2015). Studies on the Mongolian Plateau have shown that the dynamics of soil moisture significantly affect vegetation phenology, especially in dry years, where a decrease in soil moisture leads to a shorter vegetation growing season (Luo et al., 2021). Additionally, human activities such as urbanization (Li et al., 2017) have become important factors affecting phenology. Hong et al. (2023) suggested that the heat island effect and air temperature within cities jointly affect changes in urban vegetation phenology, and their contributions are almost equal. In the United States, urban warming promotes spring phenology (Meng et al., 2020) but reduces the response to temperature. Relative to those in rural areas, the urban phenology SOS is advanced, the EOS is delayed, the length of the growing season LOS is lengthened, and the temperature sensitivity of the SOS and EOS is greater in northern cities than in southern cities (Jia et al., 2021).

Many studies have investigated vegetation phenology changes at the global scale, which not only increases the understanding of mechanisms underlying phenological changes but also provides a theoretical basis for regional and local phenology characterization (Piao et al., 2019). As China has the most significant contribution to global greening (Chen et al., 2019), systematic research on the characteristics of the phonological changes in vegetation in the country and its influencing factors is highly important for understanding the carbon cycle and ecological response of terrestrial ecosystems (Du et al., 2019). However, compared to global-scale studies, there are still many gaps in vegetation phenology research in China. On the one hand, China’s complex topography and geomorphology, extensive climate zones, and significant differences between northern and southern ecosystems have led to considerable spatial and temporal heterogeneity in phenological changes. Most studies focused on typical ecological regions such as the eastern plains, Northeast China, and the Tibetan Plateau (Ge et al., 2022; Shen et al., 2011), whereas ecologically sensitive or drastically changing regions such as the north–south transition zone, urban fringes, and hilly and mountainous areas have received insufficient attention.

On the other hand, most studies have used traditional remote sensing indices, such as NDVI and EVI, for climate identification. Although these indices are valid to some extent, they cannot accurately reveal the changes in and driving mechanisms of vegetation photosynthetic functions. The introduction of new remote sensing indices, such as chlorophyll fluorescence (SIF), has provided a new opportunity to investigate the photosynthetic phenology of vegetation. However, the systematic application of SIF is insufficient in large-scale regions in China, especially because of the lack of coupled analysis with climate variables, topographic factors, and human activities (Chen et al., 2023; Zhang et al., 2021). Additionally, most studies are dominated by phenomenological descriptions and lack the integration of high-resolution multisource data and the construction of analytical frameworks for causal mechanisms, which makes predicting the response of complex ecosystems difficult (Xu et al., 2013).

Despite considerable progress in vegetation phenology research, several important gaps remain. First, most previous studies in China have relied on traditional vegetation indices such as NDVI and EVI, which primarily describe canopy greenness and may not accurately capture vegetation photosynthetic activity. Second, existing SIF-based phenology studies have mainly focused on global scales or specific regions, while comprehensive assessments of vegetation phenology across China using high-spatial-resolution SIF data remain limited. Third, the combined effects of climate factors, topography, and human activities on vegetation phenology have not been systematically evaluated within a unified analytical framework.

To address these gaps, this study employs a high-resolution HCSIF dataset (500 m) to investigate the spatiotemporal dynamics of vegetation phenology across China from 2001 to 2020. Specifically, this study aims to (1) characterize the spatial and temporal patterns of vegetation phenology, including the start of season (SOS), end of season (EOS), and length of season (LOS), across China, (2) evaluate the stability and future persistence of phenological changes using trend, coefficient of variation, and Hurst analyses, and (3) evaluate the associations between climatic factors, topographic conditions, urbanization, and vegetation phenology. The results provide new insights into vegetation photosynthetic phenology and its driving mechanisms under climate change and increasing human disturbance in China.

## 2 Materials and methods

### 2.1 Study area

**Figure 1:**
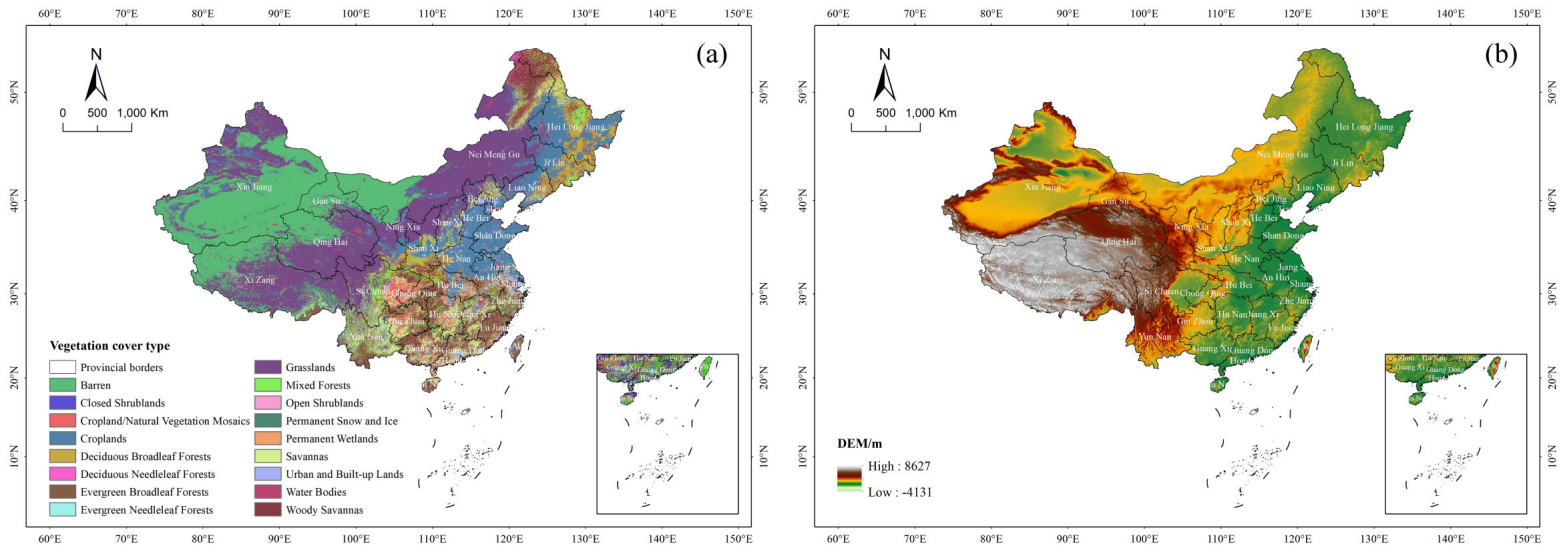
**(a)** Distribution map of major vegetation types in the study area; **(b)** the 2024 digital elevation model of the study area.

We systematically analyzed the spatial and temporal characteristics of vegetation types and their driving mechanisms in the study area of China (**Figure 1**). China is located in the East Asian monsoon region. The terrain shows a terraced distribution of high elevation in the west and low elevation in the east, transitioning from the Tibetan Plateau to the plateau hills and basins of the second terrace in the east and then to the eastern plains and coastal areas, with the land elevation ranging from the lowest elevation in the Turfan Basin to the highest elevation on Mt. Everest (8,627 m), with significant topographic relief. The climate is diverse, spanning the cold temperate zone, temperate zone, subtropical zone, and tropical zone, with an overall spatial pattern of decreasing temperature and decreasing precipitation from southeast to northwest. The hydrological conditions transition from the humid monsoon zone in the southeast to the arid inland zone in the northwest, which includes a monsoon climate, a temperate continental climate, and a highland mountain climate.

China has diverse ecosystems, ranging from broad-leaved evergreen forests along the eastern coast and deciduous broad-leaved forests and mixed forests in the Yangtze River Basin to desert grasslands, sparse grasslands, and alpine scrubs in the west, showing a spatial transition from forests to grasslands and deserts from the southeast to the northwest. According to the data on vegetation zoning and coverage in China, the following eight representative vegetation types were selected for this study: grassland, coniferous forests, broad-leaved forest, woody savannas, mixed forests, farmland, shrublands, cropland/natural vegetation mosaic, which represent the main vegetation patterns under different ecoclimatic zones and have strong ecological significance and typicality.

### 2.2 Data sources

To study the spatial and temporal dynamics of vegetation phenology and its influencing factors in China, HCSIF, meteorological, hydrological, land use, and other data were collected (**Table 1**), and each dataset is described below.

**Table 1:** Sources and applications of data.

Data	Time	Resolution	Data source
DEM	2020	500 m	https://www.gebco.net/data_and_products/gridded_bathymetry_data/
CNLUCC	2001–2020	30 m	http://www.resdc.cn/DOI
HCSIF	2001–2020	500 m	https://www.scidb.cn/en/detail?dataSetId=2498ad34919e43a79c9443f58e863f42
Prcp	2001–2020	0.1°	ERA5 Reanalysis Dataset https://www.ecmwf.int/en/forecasts/dataset/ecmwf-reanalysis-v5
SD	2001–2020	1 km	https://data.cma.cn/share/subject.html
ET	2001–2020	500 m	https://www.earthdata.nasa.gov/centers/lp-daac
SPEI	2001–2020	1 km	https://www.scidb.cn/en/detail?dataSetId=968592537239420928
Temp	2001–2020	0.1°	https://www.ecmwf.int/en/forecasts/dataset/ecmwf-reanalysis-v5
VS	2001–2020	4 km	https://www.ncei.noaa.gov/data/global-summary-of-the-day/archive/
Srad	2001–2020	4 km	
Soil	2001–2020	4 km	
PANDA	2001–2020	1 km	https://www.nature.com/articles/s41597-024-03223-1

#### 2.2.1 HCSIF data

The data used in this study included the high-resolution daylight-induced chlorophyll fluorescence dataset (HCSIF), with a temporal range from 2000 to 2022. The data were inverted using a weight stacking algorithm with TROPOMI SIF and MODIS data as input data, and the test results (*R*^2^ = 0.87, RMSE = 0.057 mW/m^2^/nm/sr) revealed that the data accuracy was high and widely used in vegetation phenology studies (Tao et al., 2024). (https://www.scidb.cn/en/detail?dataSetId=2498ad34919e43a79c9443f58e863f42).

#### 2.2.2 Meteorological and hydrological data

Meteorological conditions such as temperature and precipitation are the main drivers of vegetation phenology. The meteorological and hydrological data used in this study were mainly from ECMWF/ERA5. ERA5 is the fifth generation of atmospheric reanalysis of the global climate conducted by ECMWF, covering January 1940 to the present. In this study, average temperature and precipitation data from 2001 to 2020 with a spatial resolution of 1 km were downloaded from the Google Earth Engine (https://www.ecmwf.int/en/forecasts/dataset/ecmwfreanalysis-v5).

#### 2.2.3 Land use data

Land use data were used to visualize the effects of human activities on ecosystems, such as urbanization processes, and in this study, the MODIS land cover product based on the International Geosphere-Biosphere Programme (IGBP) classification scheme was used. This product classifies global vegetation into 17 types, from which we selected eight types for the study based on regional conditions.

#### 2.2.4 Other relevant data

To analyze the spatial and temporal characteristics of vegetation and its influencing factors, we also downloaded the 2001–2020 raster data on evapotranspiration, potential evapotranspiration, aridity index, radiation, saturated water vapor depressions, soil moisture, wind speed, elevation, and slope using the Google Earth Engine. Nighttime light data were used to classify the degree of urbanization. The prolonged artificial nighttime light dataset of China (PANDA) is an artificial nighttime- light dataset produced based on a nighttime light convolutional long-term and short-term-storage memory (NTLSTM) network (Zhang et al., 2024). The coefficient of determination (*R*^2^) and root mean square error (RMSE) of the data accuracy were 0.95 and 0.73, respectively, and the linear slope was 0.99, which indicated that the data quality of the generated products was high.

The abovementioned data file formats, as well as spatial and temporal ranges and resolutions, were all considerably different. To ensure that the data were consistent, all data with a temporal resolution of 1 year and spatial resolution of 500 m were resampled and transformed into GeoTiff format via ArcGIS, with the coordinate system of the WGS84 geographic coordinate system, to facilitate subsequent research.

### 2.3 Research methodology

#### 2.3.1 Time series reconstruction

**Figure 2:**
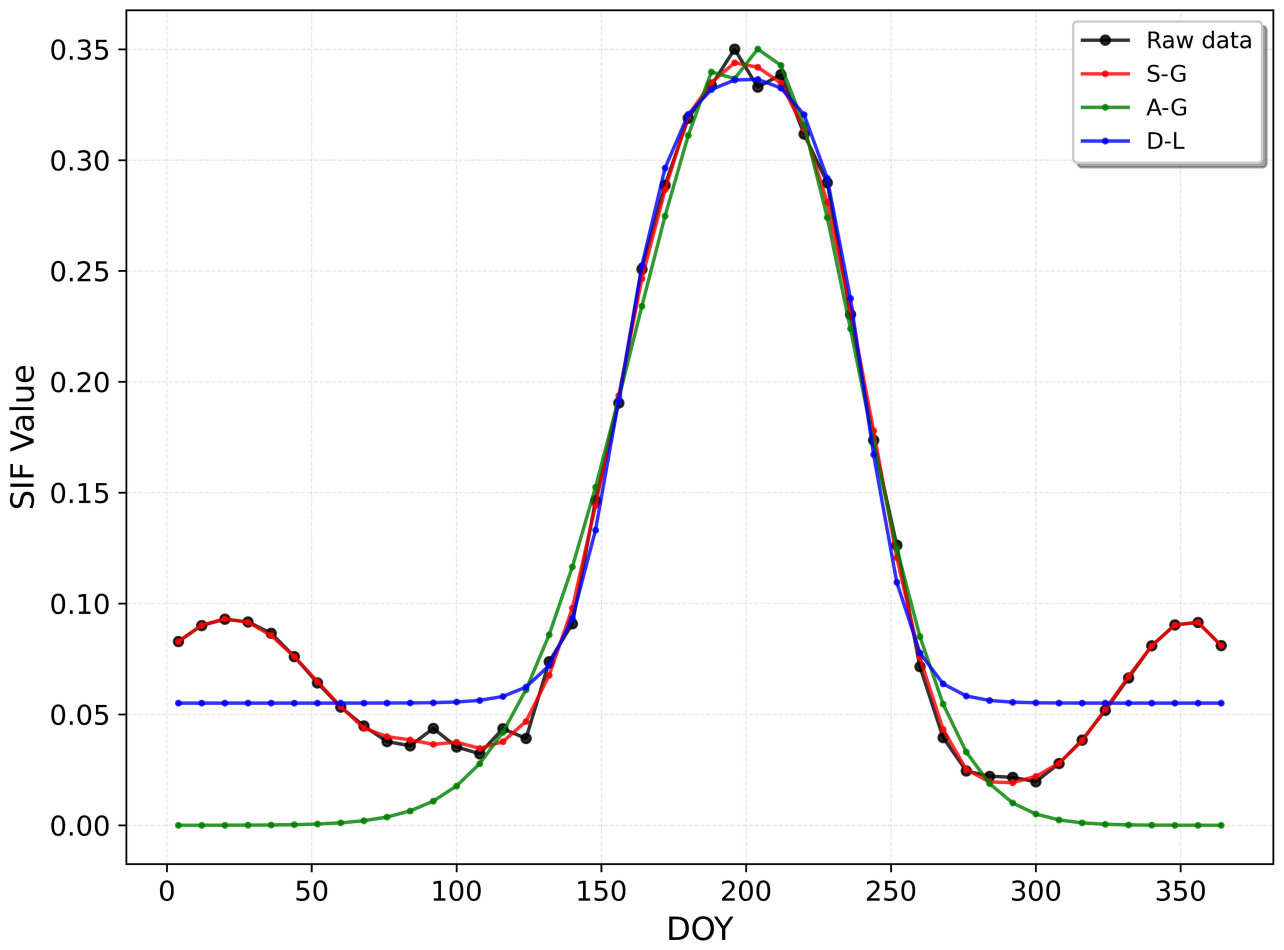
Comparison of three fit methods.

In the spatiotemporal analysis of remotely sensed vegetation phenology data, the original time series are often affected by factors such as the atmosphere, cloudiness, changes in observation angle, and atmospheric perturbations (Li et al., 2020), which lead to problems such as a lack of measurements, sharp fluctuations, and outliers in the data and are thus not conducive to the accurate extraction of phenology parameters (e.g., SOS and EOS) (Cai et al., 2017). To eliminate the interference of observation errors and restore real-time series trends, it is usually necessary to smooth and reconstruct the original time series. This paper applies three methods—S-G filtering, A-G filtering, and D-L function fitting—to fit HCSIF time series curves. The results show that the S-G filtering method produces a smoother overall curve that closely resembles the original curve, enabling a more accurate reconstruction of the vegetation index (**Figure 2**).

In this study, the S-G filtering method was used to fit the original SIF time series data. The S-G filtering method is a sliding window weighting algorithm, in which the weighting coefficients are obtained via least-squares fitting of a given higher-order polynomial within a sliding window. The new curves obtained using the S-G filtering method can retain detailed information in the process of filtering noise disturbances and can characterize the variations in long time series data more comprehensively (Chen et al., 2004). The equation used is as follows (Equation 1):

(1) $$YY_{\text{j}}^{*}=\frac{\sum_{\text{i}=-\text{n}}^{\text{n}}C_{\text{j}}Y_{\text{j}+1}}{N}$$

where *Y*_j_* denotes the reconstructed HCSIF value after S-G filtering, *Y_j_*_+_*_i_* represents the original HCSIF value within the moving window, *C_i_* is the filter coefficient, and *m* is the half-window size. The S-G filter was applied to smooth the HCSIF time series while preserving the temporal characteristics of vegetation phenology.

#### 2.3.2 Extraction and spatial and temporal characterization of phenology parameters

In this study, three key phenology parameters were extracted, including the start of season (SOS), end of season (EOS), and length of season (LOS). We referred to the methods described in other studies (Huang et al., 2019; Xu et al., 2021) (**Table 2**) and used the dynamic threshold method to reconstruct the vegetation phenology parameters. First, the time series curve of the average HCSIF was calculated from 2001 to 2020. The time when the HCSIF was first above 0.2 was considered the SOS, and the time when the HCSIF was first below 0.2 after the SOS was considered the EOS. The time difference between the two was considered the length of the growing season LOS. Following previous phenology studies, a fixed threshold of 0.2 was adopted to ensure methodological consistency across regions and facilitate comparison among different vegetation types. Previous studies have demonstrated that the 0.2 threshold can effectively identify the onset and termination of vegetation photosynthetic activity over a wide range of ecosystems. Although vegetation types differ in HCSIF magnitude, the threshold was applied to the normalized HCSIF time series, reducing the influence of absolute amplitude differences.

**Table 2:** Comparison of research methods and data types.

Reference	Threshold values	Data type	Area/vegetation type
Xu et al., 2021	Dynamic threshold (0.14–0.29)	GPP-based SIF phenology	China/grassland
Huang et al., 2019	Common thresholds (20%)	MODIS NDVI/EVI	China/crops
Yu et al., 2017	Fixed threshold (0.2)	GIMMS NDVI	Northeast China/vegetation
Guo et al., 2016	Specific thresholds (maximum annual average NDVI derivative)	AVHRR NDVI	China/winter wheat
Xu et al., 2023	Dynamic threshold (20%)	SIF, NDVI, EVI	China/bamboo forest
Wang et al., 2022	Dynamic proportion (50%)	SIF, NDVI, GPP	China/multiple vegetation zone

To analyze the spatial and temporal variation characteristics of vegetation features, one-way linear regression (slope) was performed to assess the trend of vegetation phenology from 2001 to 2020, and the significance of the trend was tested by conducting a nonparametric test (M-K) (Equation 2):

(2) $$S\text{lope}=\frac{\sum_{i=1}^{n}\left(i*y_{i}\right)-\sum_{i=1}^{n}i\sum_{i=1}^{n}y_{i}}{n\sum_{i=1}^{n}i^{2}-{\left(\sum_{i=1}^{n}i\right)}^{2}}$$

Here *i* represents the year, *n* represents the number of samples (*n* = 20), and *y_i_* denotes the value of the vegetation phenology parameter in the *i*-th year.

On the other hand, the coefficient of variation was used to measure the stability of vegetation phenology, which was calculated as follows (Equation 3):

(3) CV=Q/X

Here Q and X represent the standardized variance and mean values of the vegetation phenology parameters in the study area, respectively. According to the coefficient of variation (CV), phenological variability was classified into five levels: very low variability (CV< 0.05), low variability (0.05 ≤ CV< 0.10), moderate variability (0.10 ≤ CV< 0.15), high variability (0.15 ≤ CV< 0.20), and very high variability (CV ≥ 0.20).Finally, the future trend of vegetation phenology was examined using the Hurst index, the vegetation phenology time series were categorized into four types: strong inverse persistence (0.15 ≤ H< 0.35), weak inverse persistence (0.35 ≤ H< 0.5), weak positive persistence (0.5 ≤ H< 0.65), and strong positive persistence (H ≥ 0.65). The future development trend of vegetation phenology was further predicted, including strong persistence degradation, weak persistence degradation, anti-strong persistence improvement, anti-flak persistence improvement, anti-flak persistence degradation, anti-strong persistence degradation, weak persistence improvement, substantial persistence improvement, and no change in the nine types.

In addition, the study period (2001–2020) represents a relatively short time series for Hurst exponent estimation. Although the Hurst index can provide useful information regarding statistical persistence, its reliability may decrease when applied to short records. Therefore, the persistence results reported here should be viewed as indicative of potential trend tendencies rather than robust forecasts of future phenological trajectories.

#### 2.3.3 Biased correlation analysis of vegetation phenology

To further investigate the relationships between factors such as physical climate and climate, partial correlation analysis and Pearson’s correlation coefficient were performed to determine the correlations and test their significance. The calculation was performed using Equation 4.

(4) $$R_{xy}=\frac{\sum_{i=1}^{n}\left(x_{i}-\bar{x}\right)\left(y_{i}-\bar{y}\right)}{\sqrt{\sum_{i=1}^{n}\left(x_{i}-\bar{x}\right)}\sqrt{\sum_{i=1}^{n}\left(y_{i}-\bar{y}\right)}}$$

Here *n* denotes the year (*n* = 20), and the variables represent the objects for which the correlation analysis was conducted and denote the mean of each variable. When *R* is >0, it indicates a positive correlation between the variables; when *R* is<0, it indicates a negative correlation. The correlation is significant at *P*<0.05; the correlation is not significant at *P >*0.05.

In this study, climatic factors, topographic conditions, and urbanization were not statistically separated through variance partitioning or causal attribution models. Their relationships with vegetation phenology were evaluated independently using correlation analysis, elevation gradient analysis, and urbanization gradient analysis. Therefore, the results should be interpreted as associations rather than independent causal contributions.

## 3 Results

### 3.1 Characteristics of spatial and temporal changes in vegetation phenology in China over the past 20 years

**Figure 3:**
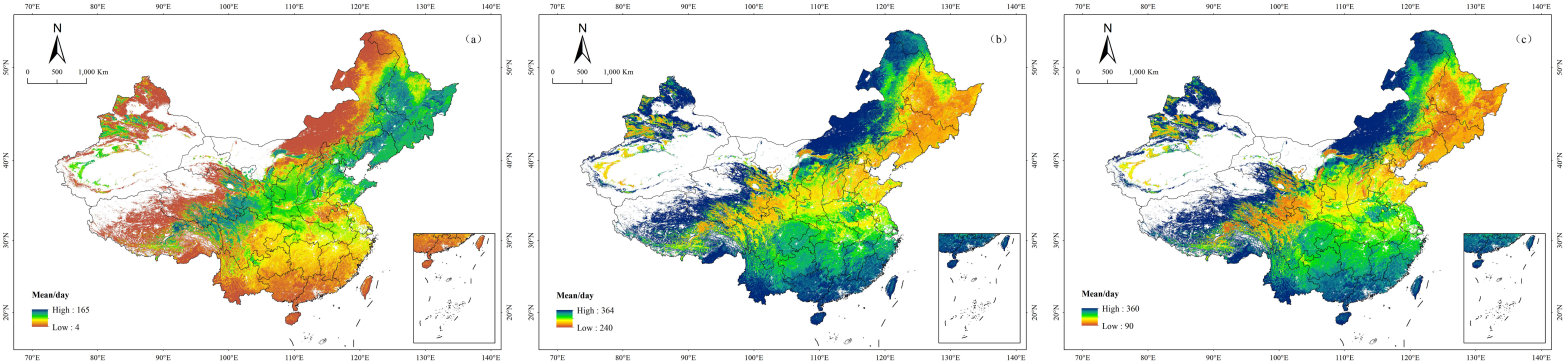
Characteristics of vegetation phenology **(a)** SOS, **(b)** EOS, and **(c)** LOS distributions.

The spatial distributions of the multiyear average SOS, EOS, and LOS of vegetation in the Chinese region from 2001 to 2020 are shown in **Figure 3**. The average number of days for the SOS and EOS in China was 59 and 321, respectively, and the LOS was 261 days. The spatial distribution of the start of the growing season from southeast to northwest was characterized as “early–late–early,” which was mainly concentrated from 4 to 160 days (accounting for 99.94% of the overall number of yuan), and the SOS in Heilongjiang, Liaoning, Jilin, and Sichuan Provinces occurred later than that in other regions. The SOS of vegetation in parts of Inner Mongoliagu, Xinjiang, Gansu, Tibet, Qinghai, and Gansu provinces was greater than that in different regions. The vegetation SOS occurred at the earliest in some areas in Gansu Province. The vegetation yellowing period was mainly concentrated from 250 to 364 days (accounting for 99.88% of the overall likeness), and the spatial distribution was characterized as “late–early–late” from southeast to northwest. The length of the growing season of vegetation was mainly between 98 and 360 days (99.93% of the overall image), and the spatial distribution was characterized as “long–short–long” from southeast to northwest, among which the LOS of vegetation in Inner Mongolia, Xinjiang, Gansu, Qinghai, Yunnan, Hainan, and Taiwan, respectively, were longer.

**Figure 4:**
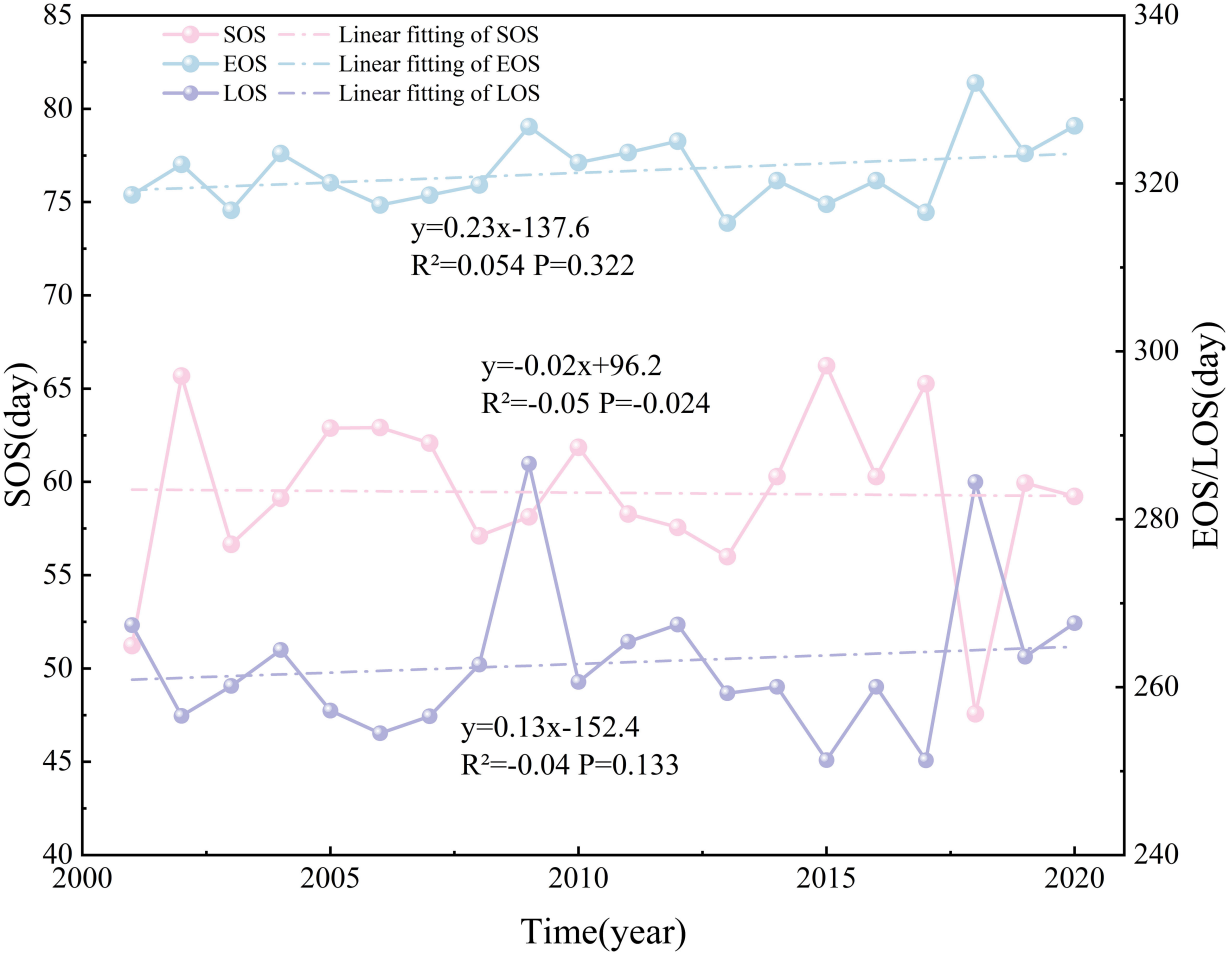
Interannual trends in vegetation phenology across China from 2001 to 2020.

**Figure 5:**
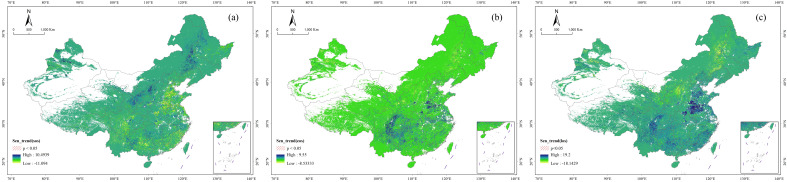
Spatial distribution and significance testing of interannual trends in vegetation phenology across China from 2001 to 2020. Figures **(a–c)** represent sos, eos, and los, respectively, and the red slashes indicate areas where the p-value is less than 0.05.

As shown in **Figures 4**, **5**, the vegetation SOS in China advanced by 0.02 days/year on average over time, and the lengths of the yellowing and growing seasons over the past 20 years showed a prominent trend. The EOS was delayed by 0.23 days/year, and the LOS was prolonged by 0.25 days/year overall. Although the magnitude of the national mean SOS trend is small, the trend was evaluated using Theil–Sen slope estimation and the Mann–Kendall significance test. The reported trend represents the average response across the entire country and masks substantial regional heterogeneity. Overall, 13.80% of the vegetation in the study area showed an advance in SOS, mainly in northern Liaoning, Hebei, Shandong, eastern Henan, northern Anhui, southern Sichuan, Guangxi, Guangdong, and Fujian, with the most significant advance in SOS at the junctions of Anhui, Henan, Shandong, and Hebei. A total of 20.10% of the vegetation in the study area showed a postponement in SOS, mainly at the junctions of Inner Mongolia, Heilongjiang, and Jilin, as well as in Xinjiang and Gansu. The development trend of vegetation in Xinjiang, southeastern Gansu, Ningxia, and northern and central Shaanxi showed a delay, with a significant trend in Xinjiang, Inner Mongolia, Gansu, and Ningxia. A total of 12.52% of the regional vegetation EOS showed a delay, mainly in southern China, with a significant delay in Sichuan, Chongqing, Guizhou, Yunnan, and the junctions of Henan, Anhui, and Shandong. The vegetation EOS is advancing in Xinjiang, southeastern Gansu, eastern Inner Mongolia, and the Jilin junction. Moreover, 24% of the regional vegetation LOS, including that in northeastern and central China, has increased, and the trend of a shortened LOS in eastern Inner Mongolia and northwestern Jilin, as well as in Ningxia, Gansu, Shaanxi, and Shanxi, is more pronounced. Additionally, 17.6% of the regional vegetation phenology LOS has increased, which is concentrated mainly in Jiangsu, Shandong, Henan, Anhui Provinces, Yunnan, Guizhou, Sichuan, and Chongqing Municipality.

**Figure 6:**
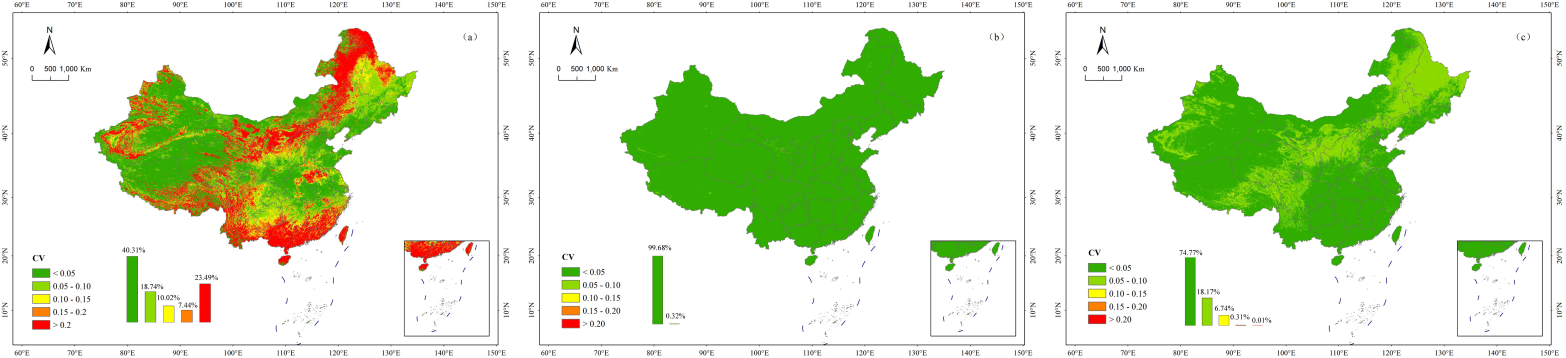
Fluctuations in vegetation phenology parameters and coefficients of variation. **(a)** Spatial distribution of the coefficient of variation for the SOS. **(b)** Spatial distribution of the coefficient of variation for the EOS. **(c)** Spatial distribution of the coefficient of variation for the LOS.

The stability statistics of the vegetation climate trends revealed that (**Figure 6**) most of the vegetation SOS in the study area is unstable, with an average coefficient of variation of 0.52, of which 23.49% are high-fluctuation areas and 7.44% are relatively high-fluctuation areas, which are mainly distributed in the northern part of the Hu Huanyong Line and the coastal areas of southern China. They are located primarily north of the “Hu Huanyong Line” and in the coastal areas of South China, including the Inner Mongolia Autonomous Region, Ningxia, Shanxi, Henan, Guangxi, Guangdong, Fujian, Hong Kong, Macao, Hainan, Taiwan, Yunnan, Sichuan, Xinjiang, Xizang, Qinghai, and other provinces, and the vegetation types are grassland and farmland. The spatial distribution of CV indicated substantial interannual variability in SOS across many regions, whereas EOS and LOS generally exhibited lower relative variability. These results describe variability patterns within each phenological metric and should not be interpreted as direct comparisons among SOS, EOS, and LOS because of their different numerical scales. In this study, CV was primarily used to assess the spatial distribution of variability within each phenological metric rather than to directly compare variability among different metrics.

The results of the persistence statistics revealed that the percentages of image elements with Hurst indices between 0 and 0.5 for the start of the growing season, the end of the growing season, and the length of the growing season were 61.81%, 70.70%, and 77.97%, respectively, which were almost randomly distributed across all areas within the study area. This result indicates that the overall changes in vegetation phenology, such as the SOS, EOS, and LOS in large areas of the study area, are characterized by development in the opposite direction to that in the past. Among them, the proportion of image elements between 0 and 0.35 was 39.99%, 45.71%, and 53.91%, respectively, indicating that the future trend of changes in the areas represented by the image elements showed a slight delay, a slight advance, and a slight shortening, respectively.

The percentages of image elements with H values of vegetation phenology parameters between 0.5 and 0.65 were 33.74%, 27.36%, and 21.46%, respectively. Among them, the SOS and LOS are distributed mainly in Northeast China and Central China, whereas the EOS is more scattered. This further indicates that these regions shall continue the past state of change in the future and show trends of advancement, delay, and prolongation. In the future, the vegetation SOS in China is likely to keep showing advancement (**Table 3**), with 14.42% of the likeness in advancement, which is primarily distributed in eastern China (Anhui, Jiangsu, Henan, Shandong, etc.) as well as in southern China (Guangxi, Sichuan, Yunnan, etc.). Delayed likeness accounts for 6.85%, with 78.73% of the likeness being located in regions that remain unchanged, and delayed likelihoods are located mainly in northern China (Inner Mongolia, Shaanxi, Ningxia, etc.). The prediction results showed that human activities have a greater effect on the vegetation EOS. The vegetation EOS shows a continuous trend of advancement in the future, with 17.81% and 2.45% likelihoods of advancement and delay, respectively. However, the same area has the opposite trend for the vegetation SOS—for example, at the junction of Inner Mongolia and Northeast China, the vegetation SOS is delayed, whereas the EOS is advanced, which leads to a shortened LOS. The vegetation LOS is dominated by persistent shortening, and the total proportion of similar areas is 29.83%, except for the Inner Mongolia Autonomous Region and Shaanxi, Ningxia, and Shanxi provinces, where the length of the growing season in the area showed persistent lengthening, and most of the vegetation in the study area showed a trend of shortening.

**Table 3:** Area statistics of persistent changes in vegetation phenology.

SOS	EOS	LOS	Future trends	
0.20%	0.01%	0.16%	Strong persistent degradation	Advance
3.39%	0.37%	2.31%	Weak persistent degradation	
2.07%	5.01%	6.91%	Anti-strength sustainability improvement	Delay
3.10%	9.63%	15.84%	Anti-weakness sustainability improvement	
6.90%	1.07%	5.81%	Anti-weak persistent degradation	Advance
3.92%	1.00%	2.74%	Anti-strong persistent degradation	
1.60%	3.07%	6.78%	Weak persistent improvement	Delay
0.08%	0.10%	0.30%	Strong Continuous improvement	
78.73%	79.74%	59.15%	Unchanged	Unchanged

**Figure 7:**
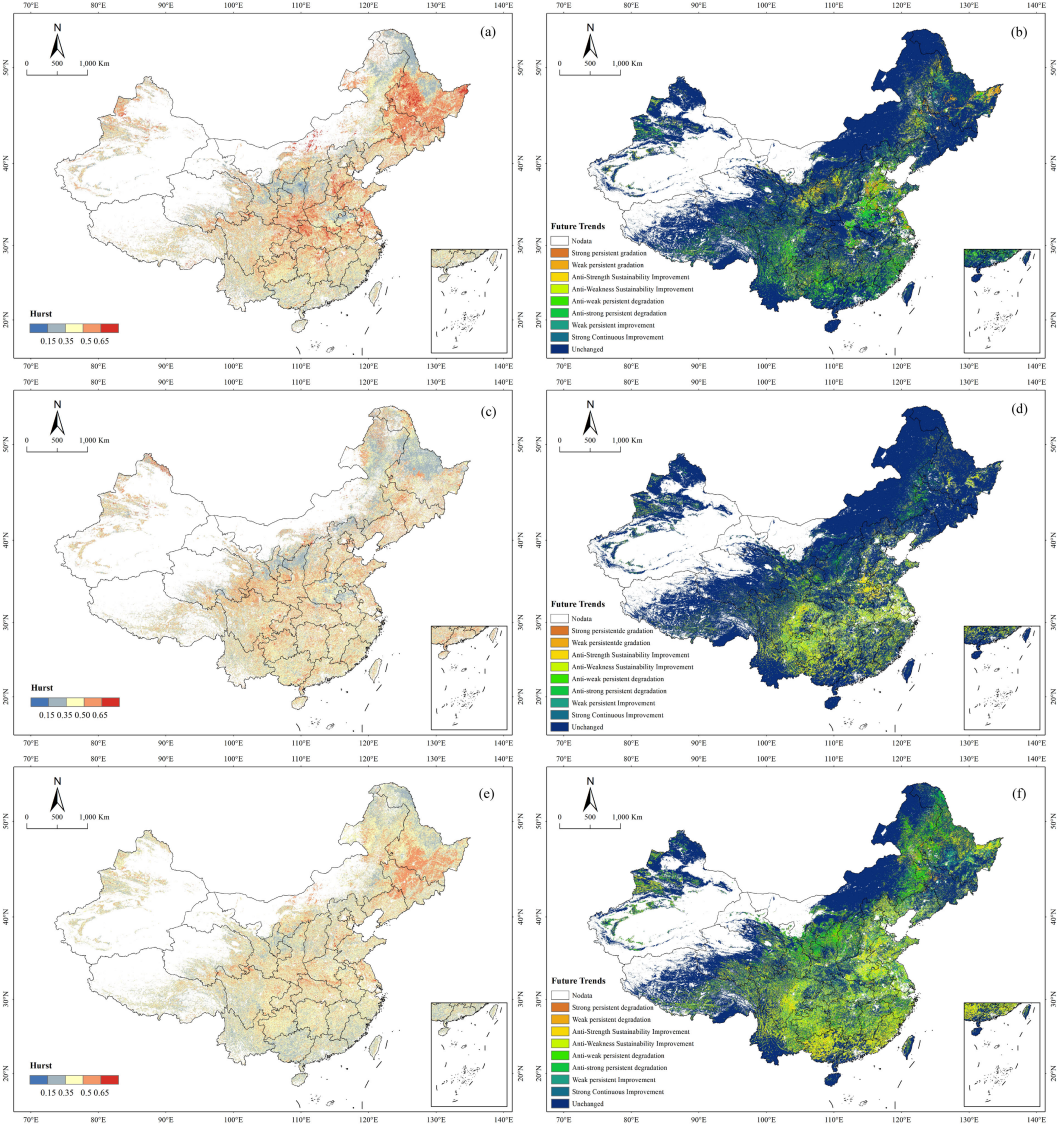
Spatial distribution of the vegetation phenology Hurst index and future trends. **(a)** Persistence analysis of the SOS. **(b)** Future trend of the SOS. **(c)** Persistence analysis of the EOS. **(d)** Future trends of the EOS. **(e)** Persistence analysis of LOS. **(f)** Future development trend of the LOS.

To analyze the persistence of the future trend of vegetation phenology in China, in this study, the parameters (Hurst) of vegetation phenology from 2001 to 2020 were calculated using the R/S analysis method at an image-by-image metric scale and combined with the results of the trend analysis of changes in Sen’s slope. We assessed the current status and future trends of the development of vegetation phenology in the study area. The results are shown in **Figure 7**. The percentages of image elements with H values of vegetation phenology parameters between 0.5 and 0.65 were 33.74%, 27.36%, and 21.46%, respectively. Among them, the SOS and LOS are distributed mainly in Northeast China and Central China, whereas the EOS is more scattered.

The proportion of pixels with Hurst indices between 0 and 0.5 for the SOS, EOS, and LOS was 61.81%, 70.70%, and 77.97%, respectively; these pixels were almost randomly distributed in all areas in the study area. This result indicates that the overall changes in vegetation phenology, such as the SOS, EOS, and LOS in large areas of the study area, are characterized by development in the opposite direction to that in the past. Among them, the proportion of image elements between 0 and 0.35 was 39.99%, 45.71%, and 53.91%, respectively, indicating that the future trend of changes in the areas represented by the image elements showed a slight delay, a slight advance, and a slight shortening, respectively. It should be noted that the Hurst exponent was estimated from a relatively short remotely sensed time series spanning 2001–2020. Although the Hurst index has been widely used to assess the persistence characteristics of environmental variables, its reliability may be affected by limited record length. Therefore, the present results should be interpreted as indications of potential future tendencies rather than deterministic predictions of future phenological dynamics. Moreover, the Hurst exponent reflects the persistence or anti-persistence of historical trends but does not explicitly incorporate future changes in climate, land use patterns, vegetation composition, or anthropogenic disturbances. Consequently, the inferred future tendencies should be interpreted with caution.

**Figure 8:**
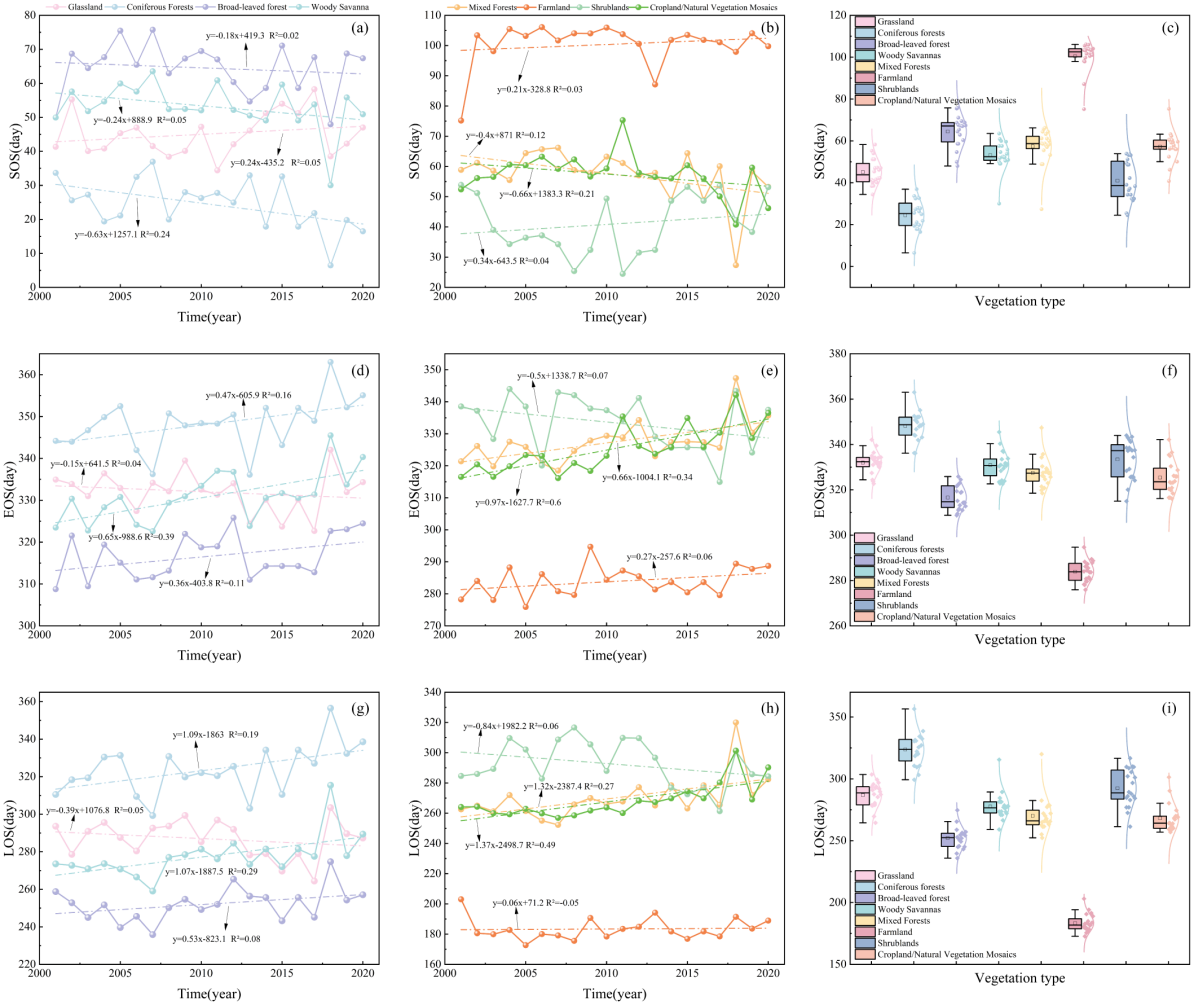
Interannual changes in the phenology of eight different vegetation types. **(a, d, g)** Interannual variation relationships of grassland, broadleaf forest, coniferous forest, and sparse vegetation with the SOS, EOS, and LOS, respectively. **(b, e, h)** Mixed forest, agricultural land, shrub, and cropland natural vegetation mosaics and SOS, EOS, and LOS interannual variability relationships, respectively. **(c, f, i)** Box plots of the interannual variations in the SOS, EOS, and LOS, respectively, for the eight different vegetation types.

To investigate the phenological changes in different vegetation types in China, we extracted the average phenological values of eight vegetation types, including broad-leaved forests, coniferous forests, mixed forests, farmlands, grasslands, sparse grasslands, croplands, natural vegetation mosaics, shrubs, etc., analyzed the long-term trends in the phenology of each type from 2001 to 2020, and plotted the trends in the changes during the phenological period over the 20 years and the fitted results (**Figure 8**; **Table 4**). The SOS of grassland, farmland, and shrubs has been delayed in the past two decades, with rates of 0.24, 0.21, and 0.34 days/year, respectively, whereas the remaining vegetation types have advanced, with coniferous forests being the most significant, with an advancement rate of 0.63 days/year and a cumulative advancement of 12.6 days in the past 20 years. Broadleaf forests show a weak trend of advancement, with a rate of 0.18 days/year and a cumulative advancement of 12.6 days in 20 years. Except for grasslands and shrubs, all vegetation types showed a delayed trend in terms of elongation at the EOS. The EOS of the grasslands and shrubs advanced by 0.15 and 0.5 days/year, respectively.

**Table 4:** Average annual trends in the phenology of different vegetation types.

Vegetation type	SOS (days/year)	EOS (days/year)	LOS (days/year)
Broadleaf forests	-0.18	0.36	0.53
Needleleaf forests	-0.63	0.47	1.09
Mixed forests	-0.40	0.66	1.32
Croplands	0.21	0.27	0.06
Grasslands	0.24	-0.15	-0.39
Shrublands	0.34	-0.50	-0.84
Woody savannas	-0.24	0.65	1.07
Cropland/natural vegetation mosaics	-0.66	0.97	1.37

In contrast, those of cropland and natural vegetation mosaic, mixed forest, and sparse grassland delayed the EOS more significantly, with rates of 0.97, 0.66, and 0.65 days/year, respectively. The LOS of grasslands and shrubs shortened at rates of 0.39 and 0.84 days/year, respectively, which was attributed mainly to the delayed SOS and advanced EOS. In addition, the LOS of the cropland, natural vegetation mosaic, and mixed forest areas significantly increased, with lengthening rates of 1.37 and 1.32 days/year, respectively, and the growing seasons increased by 27.4 and 26.4 days, respectively, over the past 20 years. Stability and sustainability statistics revealed that the vegetation EOS mainly exhibited low fluctuations and relatively stable changes, with 99.68% of the image elements in the stable area and an average coefficient of variation of 0.05. The vegetation LOS exhibited low fluctuations and relatively low fluctuations, with 74.77% and 18.17% of the image elements, respectively, and an average coefficient of variation of 0.13, indicating that the vegetation EOS and LOS changes were very stable over the past 20 years,.

### 3.2 Analysis of the climatic and environmental drivers of vegetation phenology

**Figure 9:**
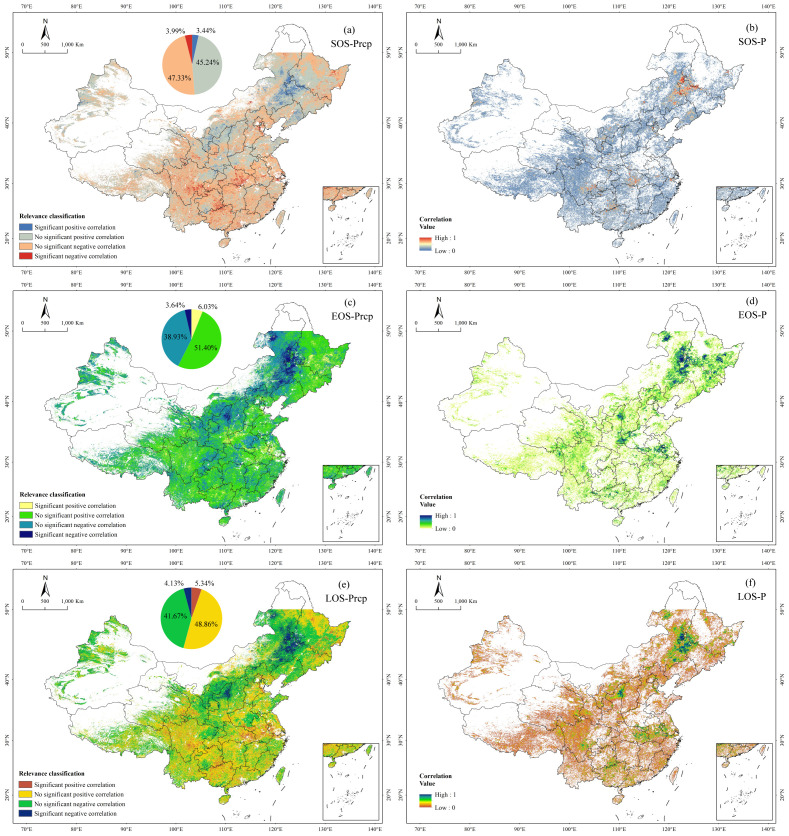
Distribution of correlation coefficients between vegetation phenology and precipitation. **(a)** Correlation between the SOS and precipitation. **(b)** Significance test of the SOS correlation with precipitation. **(c)** Correlation between the EOS and precipitation. **(d)** Significance test of the EOS correlation with precipitation. **(e)** Correlation of the LOS with precipitation. **(f)** Significance test of the LOS correlation with precipitation.

The Pearson correlation analysis method was used to analyze the vegetation phenology parameters SOS, EOS, LOS, and mean annual precipitation in China via image-by-image meta-analysis; the spatial distributions of the correlation coefficients that passed the significance test are shown in **Figure 9**. The correlation analysis with the SOS (**Figures 9a, b**) revealed that the correlation between the vegetation SOS and precipitation from 2001 to 2020 was mainly negative, i.e., the SOS increased with increasing rainfall. The negative correlation image accounted for 51.32%, which was more significant in Heilongjiang, Liaoning, and Ningxia, where the SOS advanced as precipitation increased. Correlation analysis with the EOS (**Figures 9c, d**) revealed that the relationship between the vegetation EOS and precipitation was positively correlated; the proportion of positively correlated pixels was 57.43%, and the proportion of negatively correlated pixels was 42.57%. Regarding spatial distribution, the positively correlated pixels were scattered and evenly distributed.

In contrast, the negatively correlated pixels were distributed mainly in Inner Mongolia, the three northeastern provinces, and Shaanxi, Shanxi, and Sichuan, i.e., the increase in precipitation delayed the EOS, with the most significant positive correlation occurring in Inner Mongolia. Correlation analysis with the LOS (**Figures 9e, f**) revealed that the vegetation LOS was weakly positively correlated with precipitation overall, with positive and negative pixels accounting for 54.2% and 45.8%, respectively. The areas showing positive correlations were relatively evenly distributed spatially, mainly in southern China, the three northeastern provinces, and Xinjiang, whereas northeastern Inner Mongolia and northern Shaanxi presented significant negative correlations, with the vegetation LOS continuously shortening as precipitation increased. Overall, SOS was negatively correlated with annual precipitation, whereas EOS and LOS were positively correlated. These spatial relationships suggest that regions with higher annual precipitation generally tended to exhibit earlier SOS, later EOS, and longer LOS.

**Figure 10:**
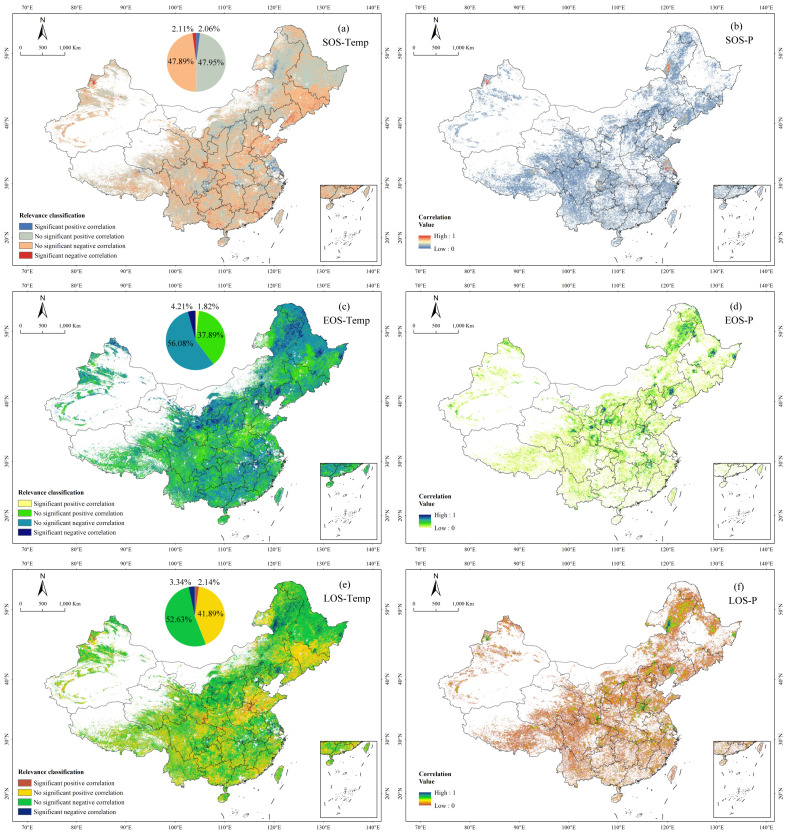
Distribution of correlation coefficients between vegetation phenology and temperature. **(a)** Correlation of the SOS with temperature. **(b)** Significance test of the SOS correlation with temperature. **(c)** Correlation of the EOS with temperature. **(d)** Significance test for the correlation of the EOS with temperature. **(e)** Correlation of the LOS with temperature. **(f)** Significance test for the correlation of the LOS with temperature.

The Pearson correlation analysis method was used to analyze the vegetation phenology parameters SOS, EOS, LOS, and mean annual air temperature in China on an image-by-image metric basis, and the spatial distributions of the correlation coefficients that passed the significance test are shown in **Figure 10**. The correlation analysis with SOS (**Figures 10a, b**) revealed that the positive and negative correlations between the vegetation SOS and air temperature from 2001 to 2020 were relatively balanced, with a weak positive correlation. Among them, the positive correlation image accounted for 50.01%, the negative correlation image accounted for 49.99%, and the spatial distribution of the two was more uniform. It was positively correlated and more significant in Inner Mongolia, Jiangsu, and Chongqing. Regions showing positive correlations indicated delayed SOS with increasing annual mean temperature, whereas other regions exhibited the opposite response, reflecting strong spatial heterogeneity in temperature–phenology relationships.

The correlation analysis with the EOS (**Figures 10c, d**) revealed that the vegetation EOS was negatively correlated with temperature, with the negative correlation image accounting for 60.29%, which was primarily distributed in Inner Mongolia, Heilongjiang, Qinghai, Ningxia, Gansu, Shaanxi, Hubei, Anhui, Jiangxi, etc., and was more significant in Inner Mongolia, Liaoning, and Gansu. This finding indicates that the increase in temperature caused the vegetation EOS of these regions to increase. The correlation analysis with LOS (**Figures 10e, f**) revealed that the vegetation LOS was significantly negatively correlated with air temperature. The positive correlation was 44.03%, and the negative correlation was 55.97%, which was substantial in Inner Mongolia, Xinjiang, Henan, and a few regions in Liaoning, i.e., the vegetation LOS decreased as the temperature increased.

**Figure 11:**
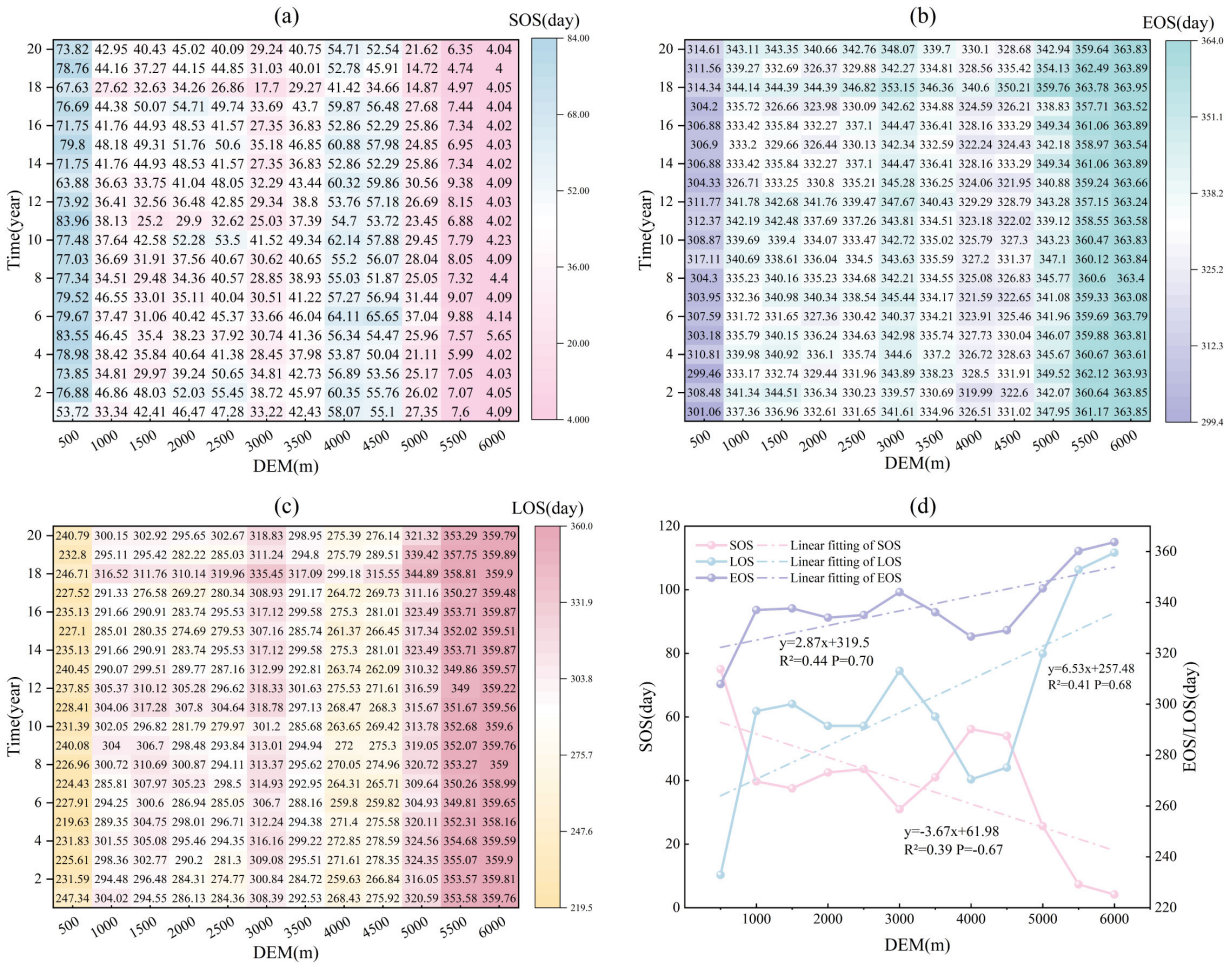
Changes in vegetation phenology with increasing altitude. **(a)** Changes in the vegetation phenology SOS with increasing altitude. **(b)** Changes in the vegetation phenology EOS with increasing altitude. **(c)** Changes in the vegetation LOS with increasing altitude. **(d)** Trends in the vegetation phenology parameters SOS, EOS, and LOS with altitude.

The spatial distribution patterns, trends, and numerical distributions of the three elements of vegetation phenology (SOS, EOS, and LOS) at different altitudes (DEM) are shown in **Figure 11**. The trends of the mean values of the phenological parameters at each altitude band are further visualized in the form of a line graph in **Figure 11d**. In this study, DEMs were divided into 12 classes (0–6,000 m, one class for each 500 m). During 20 years, as elevation increased, the SOS of vegetation advanced by 3.67 days for each 500-m increase in elevation, with significant differences (*p*< 0.01). The EOS was delayed by 2.87 days, with non-significant differences (*p* > 0.05), whereas the LOS was prolonged by 6.53 days, with no significant difference (*p* > 0.05). At 1,000–3,500 m, the vegetation SOS showed a weak trend of advancement, whereas the EOS and LOS showed a weak trend of delay and prolongation. However, within the interval of 3,000–4,000-m altitude, the vegetation SOS and EOS showed significant delays and advances, respectively, leading to a shortening of the LOS, which peaked at 4,000 m with the shortest growing season length. The vegetation SOS, EOS, and LOS in the altitude range of 4,000–6,000 m showed significant advancement, delay, and lengthening trends, respectively.

**Figure 12:**
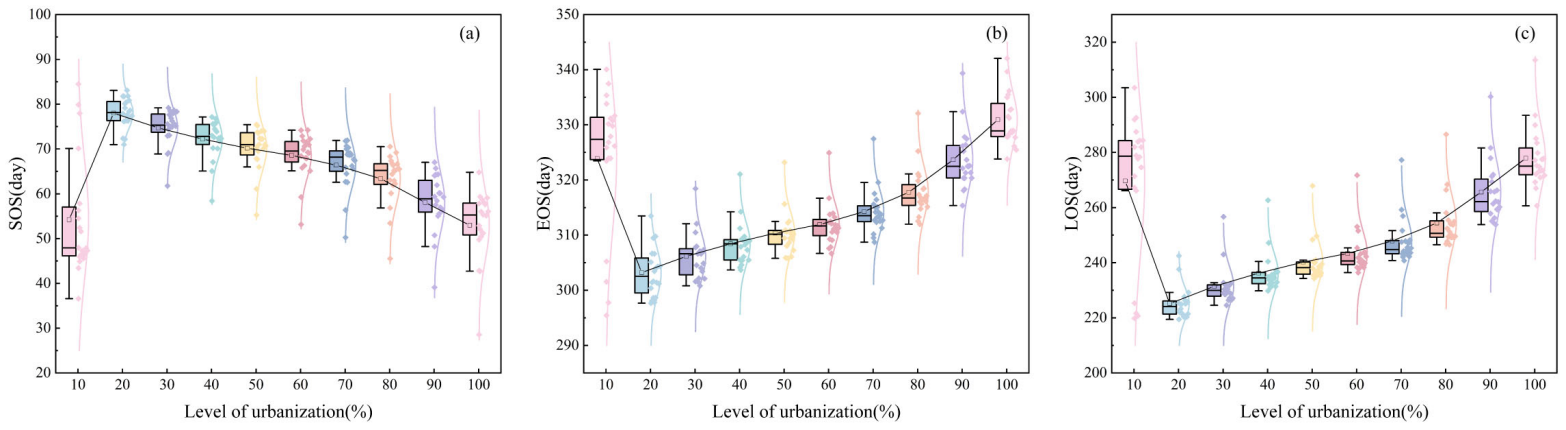
Changes in vegetation phenology with increasing levels of urbanization. **(a)** Changes in the SOS with the level of urbanization. **(b)** Changes in the EOS with increasing urbanization. **(c)** Changes in the LOS with changes in the level of urbanization.

The vegetation phenology in the study area showed different responses to the urbanization level, as indicated by the nighttime light data (**Figure 12**). Vegetation phenology was significantly correlated with the urbanization level in China over 20 years; the SOS was negatively correlated with the urbanization level, while the EOS and LOS were positively correlated. This finding indicated that the SOS advanced gradually, the EOS showed a delay, and the length of LOS increased as urbanization increased. To accurately quantify the response of vegetation phenology to urbanization, a linear regression of the dynamic urban–rural gradient in the study area was performed. For every 10% increase in urbanization during the study period, the SOS advanced by an average of 1.37 days, the EOS was delayed by an average of 2.22 days, and the length of the LOS increased by an average of 3.09 days. The rapid development of Chinese cities has had a significant effect on the spatial and temporal evolution of vegetation phenology. In this study, less than 20% of areas were defined as rural, and greater than 80% of areas were defined as urban. During the study period, the annual mean SOS in rural and urban areas was 66.13 and 55.52 days, with urban areas being 10.61 days earlier than rural areas. The EOS in rural and urban areas was 314.31 and 332.06 days, respectively, with urban areas being 17.75 days later than rural areas. The LOS in rural areas was 247.43 days, and that in urban areas was 271.79 days, i.e., the LOS was prolonged by 24.36 days in urban areas compared to that in rural areas. Therefore, a significant difference was found in the physical climate between urban and rural areas. Due to continuous urbanization, the urban ecological environment has changed significantly, which has had a profound effect on vegetation phenology. Urbanization not only changes the type of vegetation cover but also affects the environmental conditions of cities and their surrounding areas, thus indirectly or directly causing changes in vegetation phenology. As the level of urbanization increased, the greening period (SOS) of plants advanced, the end of the growing season (EOS) was delayed, and the length of the growing season (LOS) was extended.

**Figure 13:**
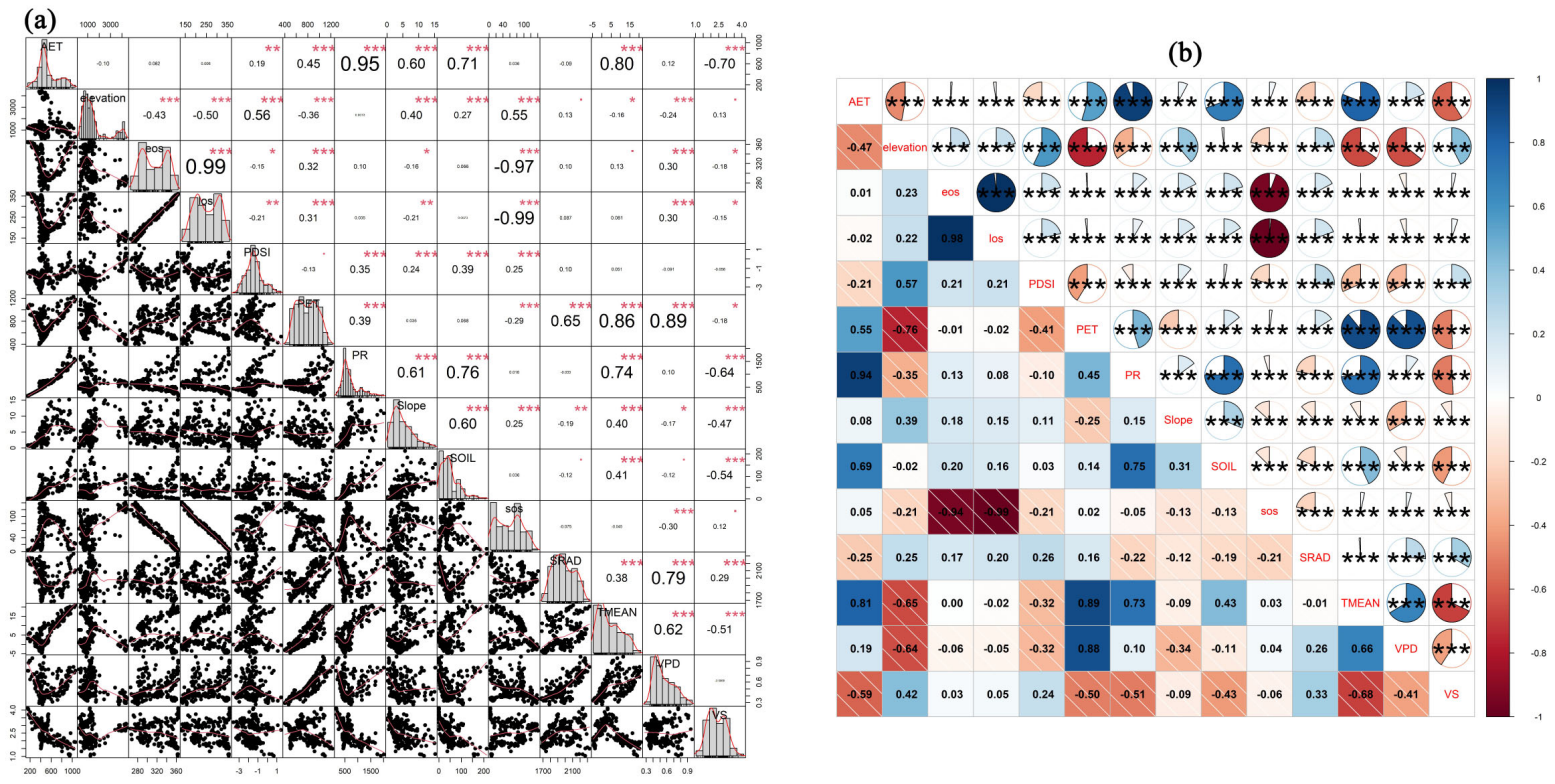
Scatterplots and fan plots of the correlation matrix. **(a)** Distribution plots are shown on the diagonal, bivariate scatterplots with fitted lines are shown on the lower left, and correlation coefficients are shown on the upper right along with significance levels. **(b)** The name of the variable is shown on the diagonal, the correlation coefficient is shown on the lower left, and the pie chart with the size of the correlation coefficient and the significance level is shown on the upper right. *** means the p-value is less than 0.001, which means the result is extremely statistically significant.

To determine the relationships between vegetation phenology parameters and environmental factors, we extracted the annual average raster data of multisource remote sensing and climate data in China from 2000 to 2020 via the GEE and constructed a correlation matrix diagram (**Figure 13**) by conducting a Pearson correlation analysis. The figure shows the correlation between the three parameters of vegetation phenology (the SOS at the greening stage, the EOS at the yellowing stage, and the LOS at the length of the growing season) and the main climatic variables (evapotranspiration (AET), precipitation (PR), mean temperature (TMEAN), saturated vapor pressure difference (VPD), and radiation (SRAD)), topographic factors (elevation and slope), soil type (SOIL), wind speed (VS), and the bivariate relationship between the drought index (PDSI). Significance levels are indicated by asterisks (*p< 0.05, **p< 0.01, and ***p< 0.001). First, there was a robust positive correlation between the EOS and LOS (*r* = 0.98, *p*< 0.001), indicating that the later the dieback period, the longer the growing season. In contrast, the LOS was significantly negatively correlated with the SOS (*r* = –0.99), i.e., the longer the growing season that started earlier, the longer it lasted. Additionally, the EOS and SOS were strongly negatively correlated (*r* = –0.94). Second, regarding the correlation of climate factors, the mean temperature (TMEAN) had a weak adverse effect on the LOS, with *r* = –0.02 (LOS), suggesting that a warmer climate shortened the duration of the growing season. The correlations of solar radiation (SRAD) with the EOS and LOS were more significant at 0.17 and 0.2, respectively, indicating that sufficient light is an essential driver for prolonging the vegetation growing season. The saturated VPD was negatively correlated with the EOS (*r* = –0.06) and LOS (*r* = –0.05), indicating that the greater the VPD, the greater the likelihood that vegetation growth would end earlier. PR was also positively correlated with the EOS (*r* = 0.13) and LOS (*r* = 0.08), reflecting the positive effect of the water supply on prolonging the growing period. AET (evapotranspiration and dispersal) was positively correlated with the effect of the SOS (*r* = 0.05) and EOS (*r* = 0.01) and negatively correlated with the LOS (*r* = –0.02), suggesting that ecosystem water use efficiency has a specific effect on phenology. This indicates that ecosystem water use efficiency also has a particular effect on phenology.

To summarize, suitable temperature, humidity, and high solar radiation can help vegetation delay the EOS and prolong the LOS, which is consistent with the trend of climate change observed in the context of global warming. Finally, the positive correlations between elevation and the EOS (*r* = 0.23) and LOS (*r* = 0.22) were relatively significant, indicating that high-elevation areas may affect vegetation phenology because of low temperatures and reduced plant respiration. The correlations between the soil type (SOIL) and climatic parameters were relatively weak. However, the significant positive correlation between SOIL and climatic factors such as TMEAN and AET indicated that soil may indirectly affect climatic conditions by regulating water and heat conductivity. The strong effect of the PDSI on the EOS and LOS (*r* = 0.21) suggested that drought stress may also be used as a reference index to study climatic changes in the region.

## 4 Discussion

### 4.1 Characteristics of vegetation phenology in the study area

The phenological metrics derived from HCSIF in this study should be interpreted as indicators of photosynthetic phenology rather than greenness phenology. Compared with NDVI- and EVI-based approaches, HCSIF is more directly linked to photosynthetic processes and carbon assimilation. Therefore, the SOS, EOS, and LOS identified in this study may better reflect the seasonal dynamics of ecosystem functioning and carbon uptake across China. This distinction is particularly important in regions where canopy greenness and photosynthetic activity are not synchronized. The interannual variation in vegetation phenology in China from 2001 to 2020 showed different degrees of fluctuation and a clear trend. The SOS advanced, and the EOS was delayed, leading to lengthening of the growing season. This finding is highly consistent with the results of several recent studies (Zhang et al., 2023). Our results suggest that temperature is an important factor influencing vegetation phenology. However, the relationship between temperature and SOS exhibited substantial spatial heterogeneity across China, indicating that phenological responses to temperature vary among regions and vegetation types. Regarding temporal characteristics, the SOS showed a trend of advancement, with an average of about 0.02 days per year, whereas the EOS was delayed, with an average of 0.23 days per year, resulting in an average annual extension of the LOS by about 0.13 days. It should be noted that the national mean SOS trend was relatively small. This value represents the average behavior across diverse climatic regions and vegetation types and may conceal substantial spatial heterogeneity. Furthermore, uncertainties associated with HCSIF observations, S-G filtering, and threshold-based phenology extraction may affect the estimated timing of SOS. Therefore, the national-scale SOS trend should be interpreted cautiously, and greater emphasis should be placed on the spatial patterns of phenological change rather than the magnitude of the national mean trend alone. The most significant climatic variation occurred in 2018, which had the earliest SOS and the most delayed EOS, and the LOS also reached the most considerable interannual difference of 35.35 days, which may have been influenced by the nationwide warmer temperatures with increased spring precipitation in that year. Shen et al. (2014) reported that temperature varied with altitude. In this study, the correlations between the SOS, EOS, LOS, and altitude increased as the altitude increased, indicating a more significant increasing trend (*R*^2^ = 0.39, *R*^2^ = 0.44, and *R*^2^ = 0.41, respectively). As the altitude increased, the SOS advanced, the EOS was delayed, and the LOS was prolonged. However, this pattern should not be interpreted solely as a direct temperature–elevation relationship. At the national scale, elevation is strongly associated with regional climate conditions, vegetation type composition, and topographic heterogeneity. Therefore, the observed phenological patterns likely reflect the combined effects of multiple environmental gradients rather than the effect of elevation alone. In particular, high-elevation regions in China are largely dominated by alpine grasslands and meadow ecosystems, which may exhibit phenological characteristics that differ from those of lowland ecosystems (Chen et al., 2020).

In terms of spatial distribution, the SOS exhibited an “early–late–early” distribution pattern from southeast to northwest, reflecting significant latitudinal differences and topographic gradient effects. This feature is pronounced in the northeast and southwest regions, where the SOS generally occurs earlier in arid and semi-arid areas such as Inner Mongolia, Xinjiang, and Qinghai, probably driven by the accelerated rate of spring warming and the accelerated melting of snow, thus affecting the vegetation phenology (Wang et al., 2018). In comparison, Heilongjiang, Jilin, and Sichuan presented relatively delayed vegetation regrowth due to the lag in spring warming. EOS was generally delayed in southern China, especially in Yunnan, Guizhou, Sichuan, and Chongqing, where warmer temperatures and delayed first frost in autumn were considered the main influencing factors (Ning et al., 2015). In contrast, in inland northwestern and alpine regions, such as Xinjiang, southeastern Gansu, and eastern Inner Mongolia, an increase in the EOS is common, reflecting the strengthening of regional aridification trends (Gao et al., 2024). For LOS, the edges of the Tibetan Plateau, Hainan Island, and Taiwan presented a longer growing season, with sufficient heat resources and relatively suitable hydrothermal conditions, essentially meeting the growth needs of plants to a large extent. However, approximately 24% of the area in the northeast and central regions showed a shortening of the LOS, especially in the eastern part of Inner Mongolia, northwestern Jilin, and the junction of Shanxi and Shaanxi, where grasslands and forests are widely distributed, and climate change and human activities (e.g., overgrazing and agricultural expansion) may be driving this change.

In contrast, the LOS is prolonged in Suzhou, Henan, Anhui, Yunnan, Guizhou, Sichuan, and Chongqing. These regions are dominated by farmland and grassland, and the vegetation types are susceptible to climatic fluctuations. Moreover, with the progress of agricultural technology, optimization of water management and other human factors, the crop growing season may also be effectively extended.

### 4.2 Analysis of factors influencing vegetation phenology

Between 2001 and 2020, the climatic periods of many vegetation types in China changed significantly, as determined by the diversification of the SOS, EOS, and LOS of different vegetation types. The driving mechanism of this change is the compound effect of multiple factors, among which climate change is the most important influencing factor. Warmer temperatures play a key role in the advancement of spring phenology, and warmer spring temperatures are among the main driving factors for the advancement of vegetation phenology (Piao et al., 2006; Richardson et al., 2013). Especially at middle and high latitudes, spring warming accelerates plant germination and leaf development, resulting in a significant advance in the return of greening in coniferous forests and other types. In contrast, there was a trend toward delayed green-up in some areas, such as grasslands and agricultural fields, which might be associated with a decrease in precipitation in winter and spring or an increase in the frequency of drought in spring (Liu et al., 2016). Drought stress inhibits the adequate supply of soil moisture and affects the normal emergence of plants, especially in precipitation-dominated regions, where the rejuvenation period is more sensitive to changes in moisture (Li et al., 2023; Wei et al., 2024).

Changes in the EOS also reflected different response mechanisms. The significant trend of a delayed EOS in arable and natural vegetation mosaics and mixed forests may be attributed to the fact that warmer temperatures slowed the senescence of plants (Jeong et al., 2011). The warmer night temperatures in the fall helped lengthen the active period of photosynthesis, thus delaying foliage senescence. The changes in the LOS synthesized the coupled effects of the SOS and EOS. The results revealed that the areas with significant LOS lengthening were mainly concentrated in agricultural fields and mixed forests, suggesting that the vegetation in these areas has more substantial production potential in the context of a warmer climate. However, the shortened LOS of grasses and shrubs reflected that the ecosystems might be in a stressful state of water–heat imbalance, especially in ecologically fragile areas where phenological changes are more sensitive to moisture changes (Fu et al., 2014).

Additionally, human activities such as changes in agricultural cropping systems, irrigation management, and water resource regulation have also indirectly affected the phenology of cropland (Fu et al., 2022; Rui et al., 2025) as have the effects of China’s eco-construction projects and land use changes on vegetation cover and the return of farmland to forests (Cai et al., 2022). Ecological projects have significantly increased vegetation cover and affected phenological timing. Especially in cultivated land and natural vegetation mosaic areas, their delayed EOS and extended LOS may also be closely related to delayed agricultural activities. Therefore, the climatic trends of different vegetation types in China are driven by multiple factors, including temperature changes, precipitation changes, and human activities, and future research on climatic changes should further strengthen the analysis of the response mechanisms to climate extremes and land use changes.

We analyzed the drivers of vegetation phenology changes in the Chinese region. We examined in detail the effects of temperature and precipitation on vegetation phenology in the area (Pereira et al., 2016). It should be noted that the precipitation variable used in this study represents annual mean precipitation rather than seasonal precipitation. Therefore, the observed relationships primarily reflect large-scale spatial associations between hydroclimatic conditions and vegetation phenology. Because vegetation phenology is often regulated by precipitation during specific periods, such as spring precipitation for SOS and autumn precipitation for EOS, the mechanisms underlying these relationships should be interpreted with caution. In addition, this paper does not examine other factors in detail, but they also influence changes in vegetation—for example, light radiation (Xiang et al., 2024), cumulative temperature, CO_2_ concentration, and drought (Ge et al., 2021) affect vegetation. During urbanization, changes in vegetation cover type (Zhang et al., 2020) can also cause changes in phenology—for example, the seasonal rates of change in vegetation phenology in Beijing are 1.3 and 1.1 days/year at the beginning and end of the season, respectively (Zhang et al., 2022). Moreover, light pollution and air pollution may also affect vegetation phenology by influencing the photosynthesis of vegetation (Zhu et al., 2024). Additionally, errors in land cover types of unnatural vegetation and climatic datasets may also affect changes in vegetation phenology. Given these shortcomings, we consider the following three areas for future studies. First, in this study, since only the HCSIF dataset is quite homogenous and the period is only 2001–2020, future studies should combine other related data, such as the LAI, GPP, and NPP, and integrate series products with longer time series and higher resolutions to further assess the response relationship between vegetation phenology and climate change. Second, the results of this study are based on remotely sensed data and need to be validated in the future by combining field monitoring and process-based ecosystem modeling. Third, the effects of climatic factors and human activities on vegetation phenology in the region need to be further investigated.

### 4.3 Integrated controls on vegetation phenology across China

The spatial heterogeneity of vegetation phenology across China is not controlled by a single environmental factor but rather reflects the combined effects of vegetation distribution, climate gradients, topographic conditions, and human activities. Climate gradients provide the primary environmental constraints on vegetation growth, while elevation modifies local temperature and moisture conditions, thereby influencing phenological timing (Jiao et al., 2020; Zhang et al., 2022). Furthermore, elevation itself is not a direct ecological driver but rather an integrated indicator of multiple environmental gradients, including temperature, precipitation, radiation conditions, vegetation composition, and human disturbance intensity (Qiu et al., 2013). Therefore, the observed elevational patterns should be interpreted as integrated responses to coupled environmental factors rather than as the independent effect of elevation alone. At the same time, different vegetation types exhibit distinct physiological responses to climatic conditions, resulting in considerable variability in SOS, EOS, and LOS among ecosystems (Wu et al., 2016). The observed “early–late–early” pattern of SOS and the corresponding “late–early–late” pattern of EOS from southeastern to northwestern China can be understood as the result of interactions among hydrothermal conditions, vegetation composition, and topographic heterogeneity. Southeastern China is characterized by warm and humid climates, dense forest vegetation, and relatively low elevations, which generally favor earlier spring activation and prolonged photosynthetic activity. In contrast, arid and semi-arid regions in northwestern China are more strongly constrained by water availability, leading to different phenological responses. Furthermore, urbanization may locally modify thermal environments through urban heat island effects, further contributing to regional phenological variation (Ji et al., 2023; Yang et al., 2025). Therefore, the spatial patterns of vegetation phenology observed in this study should be viewed as the outcome of multiple interacting environmental and anthropogenic drivers rather than the influence of any single factor.

### 4.4 Limitations and future perspectives

Although this study evaluated the relationships between vegetation phenology and climatic factors, topography, and urbanization, it did not explicitly quantify the independent contribution of each factor. The analyses were primarily based on Pearson correlation, elevation gradient analysis, and urbanization gradient analysis, which can reveal statistical associations but cannot fully separate the coupled effects among climate, terrain, vegetation type, and anthropogenic influences—for example, elevation is closely related to regional temperature and precipitation patterns, while urbanization may simultaneously alter local temperature, moisture, land cover, and management practices. Therefore, the reported relationships should be interpreted as potential environmental associations rather than strict causal contributions.

Another source of uncertainty originates from the use of a fixed threshold (0.2) for SOS and EOS extraction. Although this threshold has been widely adopted in previous phenological studies and facilitates comparisons among regions and vegetation types, it remains an empirical parameter rather than a universally applicable physical threshold. Different vegetation types may exhibit distinct HCSIF amplitudes and seasonal dynamics, which could introduce systematic biases in the estimation of absolute phenological dates. While the use of normalized HCSIF series reduces part of this uncertainty, the extracted phenological metrics may still be sensitive to threshold selection. Future studies should evaluate the robustness of phenological estimates using multi-threshold or adaptive-threshold approaches. Future studies should also employ more rigorous attribution frameworks, such as variance partitioning analysis, structural equation modeling, random forest, or SHAP-based feature attribution methods, to quantify the relative importance of climatic, topographic, and anthropogenic drivers. Integrating these methods with long-term field observations and process-based ecosystem models would further improve the mechanistic understanding of vegetation phenological changes across China.

Finally, the present analysis was based primarily on remotely sensed datasets and lacked direct validation from long-term ground observations. Future studies should integrate field phenological records, flux tower observations, and process-based ecosystem models to improve the accuracy of phenological estimates and better quantify the mechanisms driving vegetation responses to climate change and human activities.

## 5 Conclusions

In this study, we used high-resolution daylight-induced chlorophyll fluorescence data, land use data, nighttime landscape data, and meteorological and topographical data to analyze vegetation phenology in China in multiple dimensions, including spatiotemporal, planar, and altitudinal dimensions. Based on this, we investigated the main drivers of the interannual variability and spatial heterogeneity of phenology and the responses of climate and other influences using various methods, such as linear regression, stability analysis, and persistence analysis. We also analyzed the relationship between phenology and the level of urbanization using nighttime lighting data as a proxy to represent urbanization. Following are the important findings of the study:

1. In China, the SOS has advanced significantly by 0.02 days per year (*p*< 0.01); the yellowing stage of the EOS has been delayed by 0.23 days per year, with a non-significant difference (*p* > 0.05); and the length of the growing season of the LOS has increased by 0.13 days per year, with a non-significant difference (*p* > 0.05). Over the past 20 years, the greening stage of grasslands, farmlands, and shrubs has been postponed. In contrast, the rest of the vegetation type showed an advanced trend, most significantly in coniferous forests (0.63 days/year), and the most significant trend of a delayed SOS occurred in shrubs (0.18 days/year). A delayed trend was found in all areas except for grasslands and shrubs, where the end of the growing season advanced at rates of 0.15 and 0.5 days/year, respectively. The length of the growing season for grasses and shrubs decreased at rates of 0.39 and 0.84 days/year, with the most significant delay totaling 19.4 days over 20 years in the cropland and natural vegetation mosaics.
2. The average coefficient of variation of the vegetation SOS in the study area was 0.52, and most of the regions were unstable, with 23.49% high-fluctuation areas and 7.44% relatively high-fluctuation areas. The types of vegetation were grassland and farmland. The vegetation EOS and LOS mainly exhibited low fluctuations and relatively stable changes. The proportions of stable regions were 99.68% and 74.77%, respectively. The average coefficients of variation were 0.05 and 0.13, respectively, indicating that the changes in the vegetation EOS and LOS have been very stable over the past 20 years.
3. The proportions of pixels with Hurst indices between 0 and 0.5 for the SOS, the EOS, and the LOS were 61.81%, 70.70%, and 77.97%, respectively. Hurst analysis indicated that most regions exhibited anti-persistent characteristics, suggesting that the historical trends of advancing SOS, delayed EOS, and prolonged LOS may not be maintained in the future. However, because these inferences are based on a relatively short time series, they should be interpreted as potential future tendencies rather than definitive predictions.
4. The SOS was positively and negatively correlated with temperature and precipitation, respectively, whereas the EOS and LOS were negatively correlated with temperature and positively correlated with rainfall. When the temperature increases, the SOS is delayed, and the EOS and LOS are advanced and shortened, respectively; when the precipitation increases, annual mean precipitation was negatively correlated with SOS and positively correlated with EOS and LOS in many regions.
5. Elevation showed notable associations with vegetation phenology. SOS exhibited a significant advancing trend with increasing elevation, whereas EOS and LOS tended to show delayed and prolonged patterns. Vegetation phenology was significantly correlated with the degree of urbanization, i.e., with the intensification of urbanization, the SOS, EOS, and LOS gradually advanced, got delayed, and lengthened, respectively. The SOS was negatively correlated with the degree of urbanization, whereas the EOS and LOS were positively correlated.

Overall, the spatial variation of vegetation phenology across China reflects the combined influence of climatic gradients, vegetation type distribution, topographic heterogeneity, and human activities. These factors interact to regulate vegetation photosynthetic dynamics and jointly shape the observed patterns of SOS, EOS, and LOS at the national scale.

## Data Availability

The raw data supporting the conclusions of this article will be made available by the authors, without undue reservation.
